# Diversity and distribution of CYP gene family in Bactrian camel

**DOI:** 10.1007/s10142-017-0571-y

**Published:** 2017-09-12

**Authors:** Surong Hasi, Jirimutu Yao, Siriguleng Yu, Yanan Tian

**Affiliations:** 1 Key Laboratory of Clinical Diagnosis and Treatment Technology in Animal Disease, Ministry of Agriculture; College of Veterinary Medicine, Inner Mongolia Agricultural University, Hohhot, 010018 China; 2 College of Food Science and Engineering, Inner Mongolia Agricultural University, Hohhot, 010018 China; 3 Department of Veterinary Physiology and Pharmacology, Texas A&M University, College Station, TX 77843 USA

**Keywords:** Bactrian camel, CYP gene family, Comparative genomic analysis, Metabolic pathways

## Abstract

Cytochrome P450 (CYP) enzymes belong to a superfamily of monooxygenases which are phase I enzymes responsible for the first pass metabolism of about 90% of drugs in animals. However, these enzymes are often polymorphic and metabolism of the same drug in different species or different individuals is influenced by genetic and non-genetic factors. Bactrian camels are capable of survival in harsh living environments, being able to consume diets that are often toxic to other mammals and can tolerate extreme water and food deprivation. The aim of this study was to investigate whether the Bactrian camel’s special metabolic pathways and unique detoxification capabilities are attributable to particularities of the CYP gene family. The Bactrian camel’s whole genome sequencing data were systemically analyzed and annotated, and then, CYP gene family was searched from the whole protein database and compared with CYP gene families of cattle, horse, chicken, and human. The total of 63 CYP gene copies were found in Bactrian camel’s whole genome and were classified into 17 families and 38 subfamilies. Among them, 9 multi-gene families were found, and CYP2, CYP3, and CPY4 have 27, 6, and 7 subfamilies, accounting for 43, 10, and 11% in camel CYP gene, respectively. In comparison with cattle, chicken, horse, and human, the distribution of CYP gene subfamilies in camel is different, with more CYP2J and CYP3A copies in the Bactrian camel, which may contribute to the Bactrian camel’s specific biological characteristics and metabolic pathways. Comparing to the cow, horse, chicken, and human CYP genes, the distribution of CYP gene subfamilies is distinct in the Bactrian camel. The higher copy number of CYP2J gene and CYP3A gene in Bactrian camel may be the important factors contributing to the distinct biological characteristics and metabolic pathways of Bactrian camels for adaptation to the harsh environments.

## Introduction

Bactrian camel (*Camelus bactrianus*) is a domesticated, even-toed ungulate native to the steppes of Central Asia. The Bactrian camel was used as an important means of transportation on the “Silk Road” by northern nomads and functioned as the “ship in the desert” making contributions to promote the exchange of trade and culture between the East and West. Unique behavioral, anatomical, and physiological characteristics of Bactrian camel allow it to survive in environments with scarce water by reducing the metabolic rate and regulating body temperature. For example, body temperature of dromedary camel in response to the environment, the so-called adaptive heterothermy, can fluctuate up to 8 °C (34 to 41.7 °C) (Bouâouda et al. 2014). In addition, Bactrian camels can consume a variety of the plants including Asteraceae and Chenopodiaceae which have a high salt content. They can also thrive on desert plants, such as the herbaceous, bush, and cacti which are rich in fiber but contain very little protein. They even feed on *Cynanchum* and *Stellera* which are toxic to other mammals. Camels can tolerate a high dietary intake of salt, consuming eight times more than cattle and sheep, yet they do not develop diabetes or hypertention (FAO of United Nations. 1994; Vito et al. 2009). A recent genomic study indicates that 2730 genes of the Bactrian camel evolve significantly faster than those of the cow and human and are frequently involved in metabolic pathways (Jirimutu et al. 2012). The lipid and arachidonic acid metabolism pathways are unique and especially the arachidonic acid which is closer to a bird (Avian). These physiological characteristics indicate that Bactrian camels possess distinct ability for the metabolism and disposition of the toxicants/drugs, suggesting that the Bactrian camel has a highly evolved detoxification system including the cytochrome P450 system.

Cytochrome P450 is a large family of mixed functions of monooxygenase which play critical roles in the first pass metabolism of endobiotics and xenobiotic compounds, many of which are toxic and carcinogentic (Sheweita 2000; Tom and Amy 2007). Cytochrome P450 (CYP) enzymes are polymorphic and CYP gene expressions are regulated at genetic and epigenetic levels. The gene expression and enzymatic activity of the CYPs are also known to be influenced by non-genetic factors such as age, gender, nutritional status, and drug-drug interactions (Umamaheswaran et al. 2014; Marcella et al. 2004). Among the CYP enzymes identified, CYP1A2, CYP2C9, CYP2C19, CYP2D6, CYP3A4, and CYP3A5 in combination are responsible for over 90% of the metabolism of drugs and xenobiotics (Tomoko et al. 2015). These enzymes mainly are expressed in the liver and they are also found in the small intestine, lung, kidney, and placenta (Virginie and Maria 2013).

The CYP genes of camel, cow, horse, chicken, and human were comparatively analyzed based on the annotated protein sequences and the newly available camel DNA sequence information (Jirimutu et al. 2012), to understand the uniqueness of CYP genes in the Bactrian camel. Our results revealed the unique CYP gene family in the Bactrian camel which may be important for its ability of xenobiotic detoxification and adaptation to the harsh desert environment.

## Methods

### Sampling and DNA extraction

The ear notch samples were collected from the wild Bactrian camel in the Mongolian Wild Camel Protection Area at Altai province, Mongolia, and from the domestic Bactrian camel in Alshaa Aimag in Inner Mongolia, China, respectively. The sample collection procedure was approved by the Camel Protection Association of Inner Mongolia for the control and supervision of experimental camels. DNA was extracted from the ear tissues based on the classical phenol-chloroform isolation methods (Rohland and Hofreiter 2007). Briefly, the tissue samples were homogenized and treated with proteinase K, then the phenol-chloroform was added to the homogenates to separate the DNA from the rest of cellular extract. The RNase was then added to the aqueous phase containing DNA to eliminate RNAs. The DNA was precipitated.

### Sequencing and establishment of the database

A total of 198.6 Gb usable sequences (90-fold coverage) of wild Bactrian camel were obtained by using three different second generation sequencing technologies on Illumina/Solexa’s GAII, Life/APG’s SOLID, and Roche/454’s GS FLX platforms. Eighty-gigabyte sequences (36-fold coverage) of domestic Bactrian camel were obtained by using Solexa and SOLID platforms. Two paired-end/mate-pair sequencing libraries were constructed with insert sizes of 500 bp and 3 kb. Based on the integrity of the sequence data and genetic purity of the species, we used mainly the data from the wild Bactrian camel to systematically analyze the CYP gene family.

### Contig splicing

The original raw reads were quality-controlled, filtering out the low quality reads. Overlapping sequence information was used to assemble the short reads, and Bruijn map data structure was used to construct the contigs. The scaffold was progressively assembled with contigs using SOAPdenovo software (http://soap.genomics.org.cn/soapdenovo.html).

### Gene prediction and functional annotation

First, the ab initio based program (Altschul et al. 1997) was used to predict the genes of the Bactrian camel using the whole genome of a wild Bactrian camel (Jirimutu et al. 2012). Next, a homology-based method (Kankainen et al. 2012) was used to annotate the whole genome of the wild Bactrian camel. Finally, the ab initio-based results and homology-based results were integrated using the Evidence Modeler technique (Burge and Karlin 1997). The proteins were annotated by using InterProScan (v4.3) (Quevillon et al. 2005) (http://www.ebi.ac.uk/Tools/pfa/ iprscan/) protein structure domain analysis software from EBI (European Bioinformatics Institute). The protein coding gene dN/dS ratio was estimated by the Phylogenetic Analysis by Maximum Likelihood (PAML) package for the camel and its closest cattle orthologs, taking the human ortholog as an outgroup. The metabolic pathways were analyzed by KEGG automated annotation software KAAS (Stanke et al. 2004) (http://www.genome.jp/tools/kaas/). Using Blast (Basic Local Alignment Search Tool) to compare with the public databases and gene functions, all protein sequences were annotated and the EC number, KEGG number, and COG number were obtained.

### Analysis of the CYP genome of the Bactrian camel

All CYP structure domains of the wild Bactrian camel were obtained using the iProclass (http://pir.Georgetown.edu/iproclass/) on the annotated protein sequences. The best hits with coverage over 70% CYP-related gene family proteins were selected from wild Bactrian camel Blast KEGG (http: //www.genome.jp/kegg/), then the wild Bactrian camel CYP gene family was annotated utilizing the Blast KEGG protein database and NCBI nr (non-redundant) database (http: //www. ncbi.nlm. nih.gov/).

### Annotation of genes of other species

The total annotated protein structure information of human, horse, cow, and chicken was obtained from ENSEMBL (http://www.ensembl.org/) database and used for identification of the protein structure domain of CYP proteins, and KEGG and nr databases were blasted for annotation of the CYP gene family.

### Statistical methods

The accelerated evolutionary genes of metabolic pathways in the Bactrian camel and cow were statistically compared by using Z-statistics and permutation.

## Results

### Splicing and annotation of Bactrian camel genome

The assembly was performed by SOSPdenovo genome assembler which generated 120,352 scaffolds, and among them, 13,544 scaffold were longer than 1000 bp and 3453 scaffold were longer than 10 kb. The scaffold N50 with length greater than 1000 bp is 2 Mb and the total length is 2,005,940 bp. The GC content of the assembled scaffold was 41.28%.

In annotating the genome of wild Bactrian camel, a total of 4756 unique genes were predicted, and among them, 3774 genes lacked GO annotation and 4192 genes were without paralogs. In total, more than 1100 syntenic blocks were identified in the camel genome, which cover 12,965 orthologous camel genes and about 60% scaffolds (Jirimutu et al. 2012).

### Analysis of evolutionary rate of Bactrian camel genome

The average evolutionary rate between the Bactrian camel and cow protein coding genes was compared by using PAML to estimate the protein coding gene dN/dS value of the Bactrian camel and cow. In the genome of the Bactrian camel, 2730 genes were found to evolve at a faster rate than cow (FDR < 0.05). Through KEGG analysis, these genes were found to be enriched in the metabolic pathways (Table 1), suggesting that these genes have been evolved in adaptation to the desert environment and contribute to the unique biological characteristics of the Bactrian camel.

**Table 1:** Metabolic pathways enriched with accelerated evolutionary genes in Bactrian camel compared to cow

Pathway	Odds ratio	*P* value	FDR
Insulin signaling pathway	2.07	5.97E-05	9.05 E-04
Adipocytokine signaling pathway	1.87	7.03 E-03	3.12 E-02
mTOR signaling pathway	2.55	4.01 E-04	3.85 E-03
MAPK signaling pathway	1.57	1.51 E-03	9.88 E-03
Valine, leucine, and isoleucine biosynthesis	4.58	1.32 E-03	9.28 E-03
Lysine degradation	2.24	1.62 E-03	1.01 E-02
Citrate cycle (TCA cycle)	3.00	9.98E-05	1.20 E-03
Fructose and mannose metabolism	2.80	2.20 E-04	2.35 E-03
Glycerolipid metabolism	1.96	1.21 E-02	4.98 E-02
Type II diabetes mellitus	2.47	8.44 E-04	6.48 E-03

Moreover, the pathways enriched fast evolving genes include insulin signaling pathway, mTOR signaling pathway, and type II diabetes-associated pathways, all of which are important for the regulation of blood glucose. Bactrian camels have higher blood glucose than the other ruminants, and they have higher insulin tolerance. These physiological characteristics may explain the evolutionarily accelerated genes that were enriched in the metabolic pathways. In addition, genes involved in the pathways are important for the energy metabolism, Krebs cycle, lipid metabolism, and protein metabolism. This suggests that these genes play an important role for the biological characteristics of the camel’s ability to endure the lack of food, water, and their stamina. In comparison with cow, the genes involved in the MAPK signal transduction pathway were also enriched in the camel suggesting that these genes may contribute to the alertness, agility, and adaptation to the changing environment.

### Classification of Bactrian camel CYP gene families

A total of 63 genes possessed the CYP structural domains were identified by InterProScan in the wild Bactrian camel genome. Then the whole proteins of wild Bactrian camel (*Camelus ferus*) were blasted with KEGG protein database to screen out similar proteins to CYP with the best hit coverage above 70%.

Based on the statistical analysis, the Bactrian camel genome contains 63 CYP genes, which belong to 17 gene families and 38 subfamilies (Table 2). Nine of the families contain multiple members, such as the CYP2 family containing 27 members, CYP3 family containing 6 members, and CYP4 family having 7 members. However, no CYP5 family members were found.

**Table 2:** Classification and copy number of Bactrian camel CYP gene families

Family	Copies	Subfamily	Copies	Family	Copies	Subfamily	Copies
CYP1	3	CYP1A	2	CYP7	2	CYP7A	1
		CYP1B	1			CYP7B	1
CYP2	27	CYP2A	1	CYP8	2	CYP8B	2
		CYP2B	1	CYP11	2	CYP11A	1
		CYP2C	4			CYP11B	1
		CYP2D	2	CYP17	1	CYP17A	1
		CYP2E	2	CYP19	1	CYP19A	1
		CYP2F	2	CYP20	1	CYP20A	1
		CYP2J	11	CYP21	1	CYP21A	1
		CYP2R	1	CYP24	1	CYP24A	1
		CYP2S	1	CYP26	3	CYP26A	1
		CYP2U	1			CYP26B	1
		CYP2W	1			CYP26C	1
CYP3	6	CYP3A	6	CYP27	3	CYP27A	1
CYP4	7	CYP4A	1			CYP27B	1
		CYP4B	2			CYP27C	1
		CYP4F	2	CYP39	1	CYP39A	1
		CYP4V	1	CYP46	1	CYP46A	1
		CYP4X	1	CYP51	1	CYP51A	1

### Comparative analysis of Bactrian camel CYP gene copies with other animal species

The annotated protein information of cow, horse, human, and chicken was downloaded from ENSEMBL database, and InterPro (IPR) was utilized to predict the CYP functional domains of Bactrian camel. The best hit coverage over 70% was obtained with Blast KEGG database which yielded CYP-related proteins. Comparison was made with CYP genes of Bactrian camel based on the Blast KEGG and nr database.

By comparing CYP genes of Bactrian camel with the cow, horse, human, and chicken genes, we found difference in the total CYP gene copies and subfamily distribution. Bactrian camel has 63 copies of CYP genes, which resembles those of human (61) and cow (60), more than chicken (52) and less than horse (78).

**Fig. 1:**
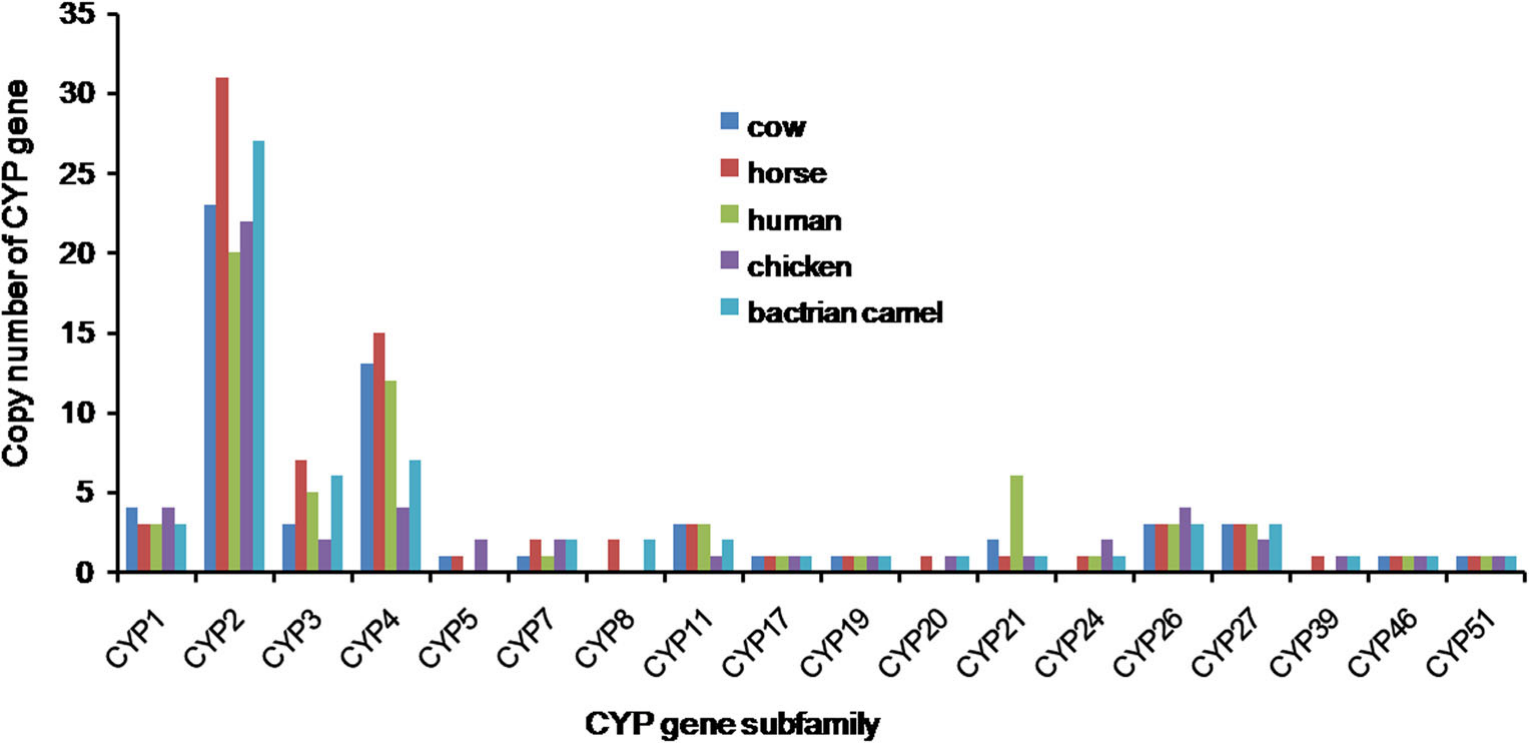
Distribution of the CYP gene family of cow, horse, human, chicken, and Bactrian camel

Among human, horse, cow, and chicken genomes, the majority of CYP genes belong to the CYP2 and CYP4 families. The numbers of genes of CYP2 family for human, horse, cow, and chicken are 20, 31, 23, and 22, respectively. The numbers of CYP4 family for human, horse, cow, and chicken are 12, 15, 13, and 4, respectively. The gene copy numbers of CYP2 and CYP4 for Bactrian camel are 27 and 7, respectively, which are ranked similarly with those of the above four species and accounted for 54% in total CYP genes (Fig. 1).

**Fig. 2:**
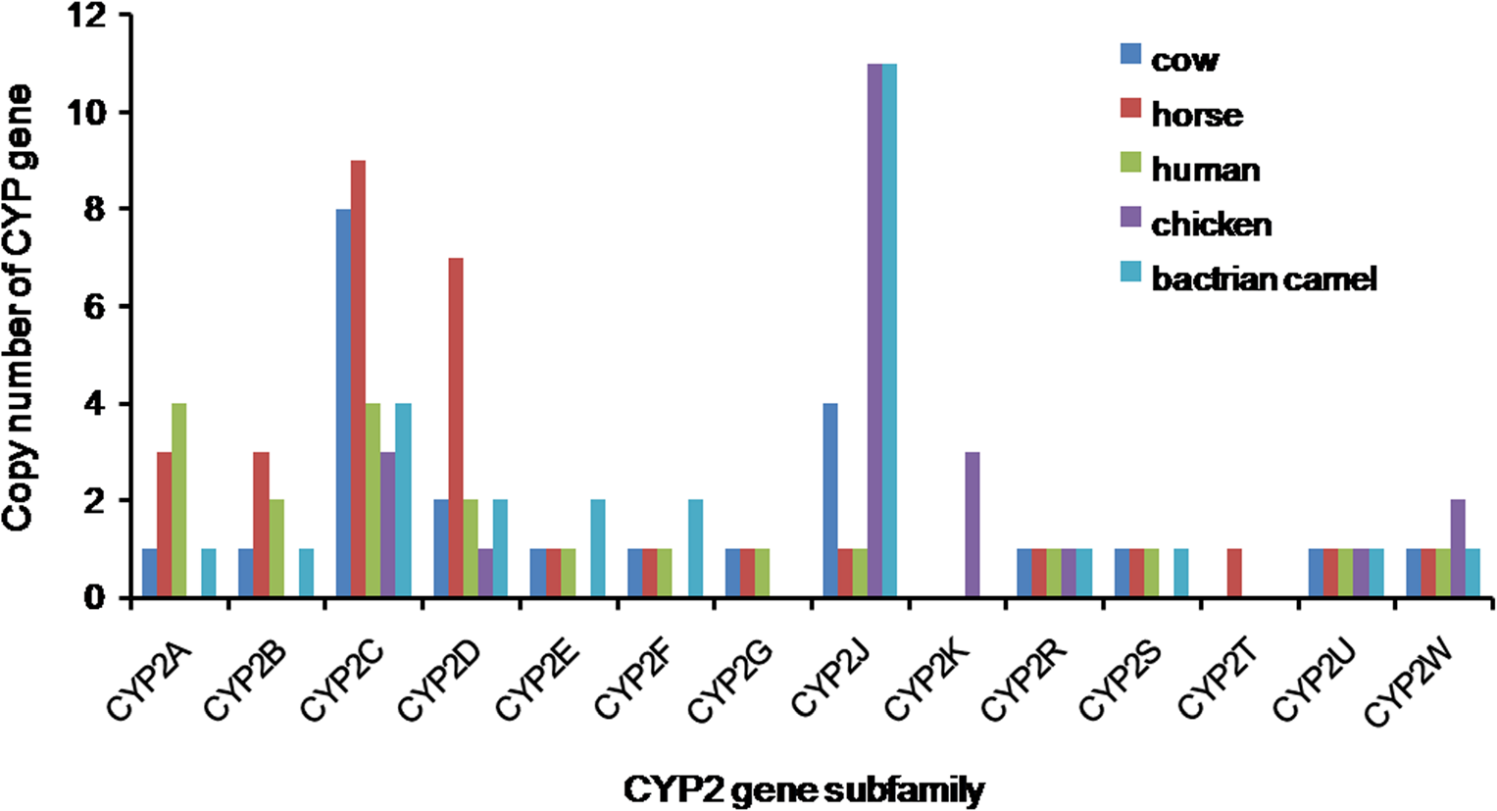
Distribution of CYP2 gene family in cow, horse, human, chicken, and Bactrian camel. Bactrian camel possesses 2, 2, and 11 copies of CYP2E, CYP2F, and CYP2J genes of the CYP2 subfamily, more than those of human, cow, and horse, especially the CYP2J gene, while chicken lacks CYP2E and CYP2F genes

**Fig. 3:**
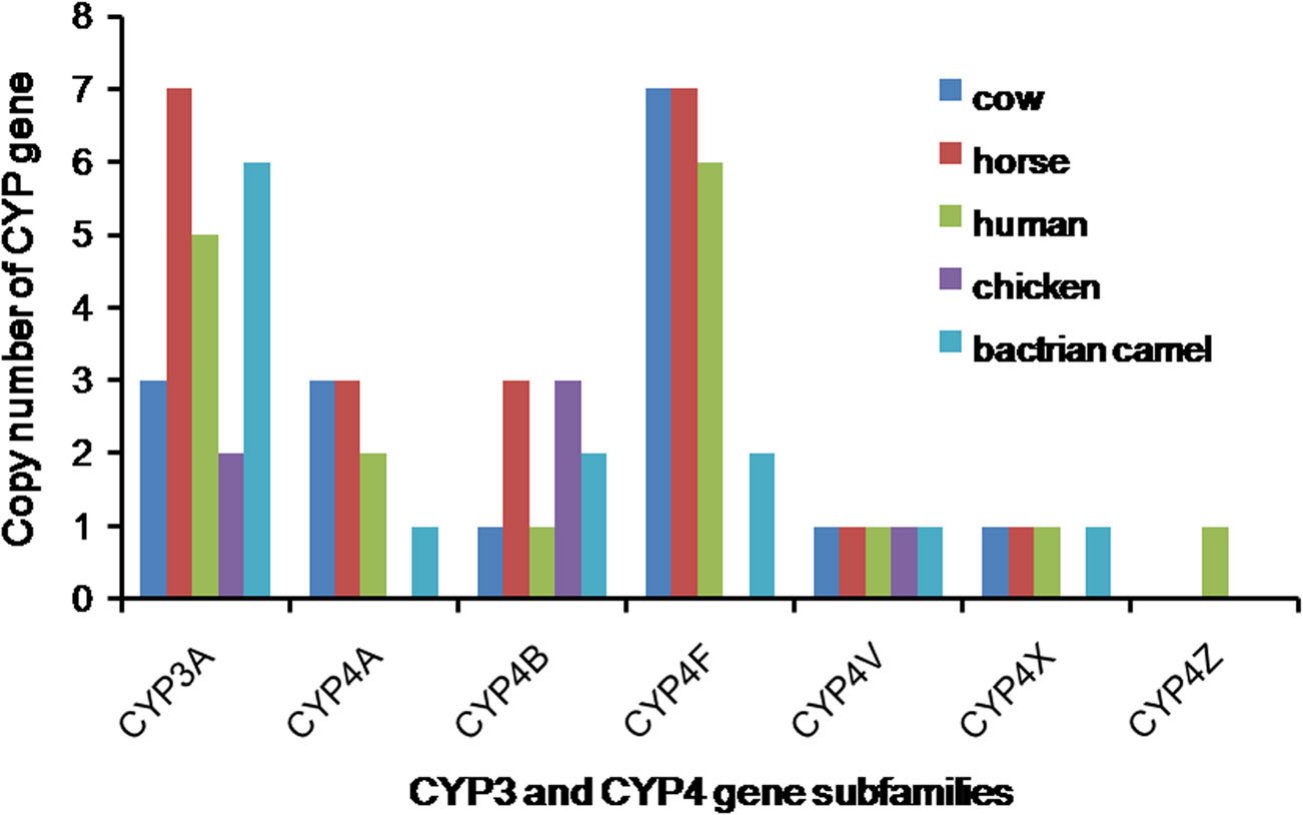
Distribution of CYP3 and CYP4 gene subfamilies in cow, horse, human, chicken, and Bactrian camel. Bactrian camel CYP3A gene copies are similar to those of horse and human, and much more than cow and chicken. Bactrian camel has only 1 copy of CYP4A gene and 2 copies of CYP4F gene which are less than those of human, horse, and cow

The numbers of gene copies distributed in the family of CYP2, CYP3, and CYP4 were further analyzed respectively and shown in Figs. 2 and 3. Bactrian camel possesses 2, 2, and 11 copies of CYP2E, CYP2F, and CYP2J genes of the CYP2 family. For CYP2E and CYP2F, the Bactrian camel has more copies than human, cow, and horse, while chicken lacks CYP2E and CYP2F genes. Interestingly, compared with a human, cow, horse, and chicken, the camel has 11 copies of the CYP2J genes and has more than those of human, cow, and horse (Fig. 2).

CYP2J and CYP2E are responsible for transforming arachidonic acid to 19(S)HETE, which has been demonstrated to be a potent vasodilator of renal preglomerular vessels that stimulate water reabsorption (Carroll et al. 1996). On the other hand, the CYP2J2 activity is regulated by high-salt diet and suppression of CYP2J2 can lead to high blood pressure (Zhao et al. 2003). Bactrian camel lacks the CYP2G, CYP2K, and CYP2T genes, and it possesses 4 copies of CYP2C gene. The number of Bactrian camel CYP3 genes (6) is similar to that of horse (7) and human (5), but more than cow (3) and chicken (2). Compared to human, horse, and cow, the Bactrian camel has only 1 copy of CYP4A gene and 2 copies of CYP4F genes, whereas human, horse, and cow have 3, 3, and 2 copies of CYP4A and 7, 7, and 6 copies of CYP4F, respectively.

### Metabolic pathway analysis

The KEGG metabolic pathway analysis results suggest that CYP enzymes are involved in 18 different metabolic pathways which are mainly involved in the metabolism of xenobiotics and lipids. Among these, CYP enzymes are involved in six pathways of lipid metabolism and four pathways of xenobiotic metabolism. The former include fatty acid metabolism, steroid biosynthesis, primary bile acid synthesis, steroid hormone biosynthesis, arachidonic acid metabolism, and linoleic acid metabolism. The remaining eight pathways are involved in the microelements, circulatory system, and metabolic diseases. However, these CYP proteins have not been well annotated. Among the 18 annotated pathways, where CYP proteins are the major enzymes for arachidonic acid metabolism, steroid hormone biosynthesis, drug metabolism, and other xenobiotic metabolism, like in other animals, CYP1, CYP2, and CYP3 enzymes are the major monooxygenase for xenobiotic/drug metabolism in Bactrian camels.

## Discussion

Bactrian camel has large number of CYP genes, and some of them appear to have been resulted from gene duplication. Bactrian camel has 63 putative CYP genes distributed in 17 scaffolds, and 14 scaffolds have duplicated CYP genes. Certain gene family members are clustered closely together. For example, scaffold 1276.9, scaffold 1276.11, and scaffold 1276.12 have all CYP2C genes located in the scaffold 1276. Scaffold 590.50 and scaffold 590.58 have CYP1A gene and sequentially located. This arrangement is similar to rice (*Oryzasattiva* L.) genome (Zhong et al. 2002), but different from Drosophila CYP gene family where CYP gene partially clustered (Adamas et al. 2000). In addition, CYP5 family, CYP2G, CYP2K, and CYP2T subfamilies have not been identified in the Bactrian camel genome, perhaps due to the highly polymorphic nature of the CYP gene family. It has been reported that tobacco (*Nicotianababacum* L.) genome contains 44 CYP families and 263 family members, which are much more than the Bactrian camel (Xie et al. 2013).

The KEGG pathway analysis annotation indicates that the CYP genes are mainly involved in the lipid metabolism and xenobiotic metabolism. Among the enzymes, CYP2J enzymes are the key enzymes in the metabolism of arachidonic acid. Arachidonic acid is metabolized mainly by CYP2J into epoxyeicosatrienoic acids (EETs). EETs are important in the cardiovascular system. In addition, CYP2J plays a pivotal role in the xenobiotic metabolism through epoxidation, alkylation, and hydroxylation (Graves et al. 2013). Therefore, these results suggest that the higher number of copies of CYP2J genes in the Bactrian camel may play a critical role in the camel’s unique ability to metabolize and dispose the exogenous as well as the endogenous substances contributing to the unique adaptability of the Bactrian camel.

Through the analysis of copy number of CYP genes, we found that the Bactrian camel has more CYP2J genes, which is similar to chicken, but more than other three species. These results may suggest the metabolic similarity in lipid metabolism to chicken and the same morphological characteristics of the red blood cells in avian and camel (Jirimutu et al. 2012). CYP2J enzymes are mainly involved in the metabolism of arachidonic acid. In 1991, Kikuta et al. (1991) discovered that CYP2J subfamily genes are widely distributed and expressed in the cardiovascular system, kidney, and liver, playing an important role in the regulation of the cardiovascular functions. There are 11 copies of CYP2J genes in the Bactrian camel CYP gene family, and the CYP2J enzyme may play a critical role in the unique physiological features of the cardiovascular system of the Bactrian camel. Furthermore, salt is very important for the camel. It needs eight times as much salt as do cattle and sheep (FAO of United Nations. 1994; Vito et al. 2009), yet this does not seem to cause diseases in the cardiovascular system of camels, perhaps because they have more copies of CYP2J genes.

The monooxygenase enzymes involved in the xenobiotic/drug metabolism are mainly in the families of CYP1, CYP2, and CYP3. Among these enzymes, CYP3A subfamily genes are mostly expressed in the liver and are also found to be expressed in the gastrointestinal tract, kidney, prostate gland, and breast tissues. The CYP3A enzyme is involved in the metabolism of 38 categories of over 150 clinically used drugs (about 50% of the current drugs used) (Badyal and Dadhich 2001). Therefore, in human, CYP3A4 is the most important drug metabolism enzyme identified. Through analysis of genome sequences, we found the Bactrian camel, CYP3A has 6 copies of genes, and it is important to further investigate the gene expression in the camel development and tissue/organ distribution in order to further understand the unique adaptability of the camel to the adverse desert conditions.

## Conclusion

Taken together, the Bactrian camel genome contains 63 copies of CYP genes, which belong to 17 gene families and 38 subfamilies based on the annotation. They are involved in 18 metabolic pathways and play an important roles in the lipid metabolism and xenobiotic metabolism. Comparing to the cow, horse, chicken, and human CYP genes, the distribution of CYP genes in subfamilies is distinct in the Bactrian camel. The copy numbers of CYP2J and CYP3A are higher in the Bactrian camel, and these high copy numbers may be the important factors contributing to the distinct biological characteristics and metabolic pathways of Bactrian camels for adaptation to the adverse condition. Our present findings provide basis for future in depth mechanistic studies of distinct biological characteristics and adaptability of the Bactrian camel to the harsh environment.
